# Correction to: Role of Endoluminal Techniques in the Management of Chronic Type B Aortic Dissection

**DOI:** 10.1007/s00270-021-02949-4

**Published:** 2021-10-04

**Authors:** Konstantinos Spanos, Tilo Kölbel

**Affiliations:** German Aortic Center Hamburg, Department of Vascular Medicine, University Heart Center, University Hospital Hamburg Eppendorf, Martinistr. 52, 20246 Hamburg, Germany

## Correction to: Cardiovasc Intervent Radiol (2020) 43:1808–1820 10.1007/s00270-020-02566-7

The article “Role of Endoluminal Techniques in the Management of Chronic Type B Aortic Dissection”, written by Konstantinos Spanos and Tilo Kölbel, was originally published Online First without Open Access. After publication in volume 43, issue 12, pages 1808–1820, the author decided to opt for Open Choice and to make the article an Open Access publication. Therefore, the copyright of the article has been changed to © The Author(s) 2020 and the article is forthwith distributed under the terms of the Creative Commons Attribution 4.0 International License, which permits use, sharing, adaptation, distribution and reproduction in any medium or format, as long as you give appropriate credit to the original author(s) and the source, provide a link to the Creative Commons licence, and indicate if changes were made. The images or other third party material in this article are included in the article’s Creative Commons licence, unless indicated otherwise in a credit line to the material. If material is not included in the article’s Creative Commons licence and your intended use is not permitted by statutory regulation or exceeds the permitted use, you will need to obtain permission directly from the copyright holder. To view a copy of this licence, visit http://creativecommons.org/licenses/by/4.0. Open access funding enabled and organized by Projekt DEAL.

